# Association of Polymorphisms in the Promoter Region of NOS2A Gene with Primary Knee Osteoarthritis in the Greek Population

**DOI:** 10.7759/cureus.6780

**Published:** 2020-01-27

**Authors:** Andreas Leonidou, Panagiotis Lepetsos, Eustathios Kenanidis, George Macheras, Maria Tzetis, Michael Potoupnis, Eleftherios Tsiridis

**Affiliations:** 1 Orthopaedics and Trauma, Aristotle University Medical School, Thessaloniki, GRC; 2 Trauma and Orthopedics, KAT Hospital, Athens, GRC; 3 Trauma and Orthopaedics, KAT Hospital, Athens, GRC; 4 Medical Genetics, University of Athens Medical School, Athens, GRC

**Keywords:** osteoarthritis, nitric oxide, single nucleotide polymorphism

## Abstract

Introduction

A new emerging role of nitric oxide (NO) in the aetiology of osteoarthritis (OA) has been reported. Inducible NO synthase (iNOS), produced by chondrocytes, is the major source of NO in the osteoarthritic cartilage. The aim of this study is to evaluate the potential association between the -1173C/T (rs9282799), -1026 C/A (rs 2779249) and -954G/C (rs1800482) single nucleotide polymorphisms (SNPs) in the promoter of the iNOS gene (NOS2A) and the incidence of knee OA in Greek population.

Methods

Ninety-six patients with primary knee OA were included in the study along with 44 controls. Genotypes were identified using polymerase chain reaction (PCR) and DNA sequencing techniques. Allelic and genotypic frequencies were compared between patients and controls.

Results

None of the -1173C/T, -1026 C/A and -954G/C SNPs were detected in the studied population, either in patients or controls. However, another SNP was identified at the site -1056 at the promoter region, where the initial G allele was substituted by the T allele. Interestingly, the TT genotype was completely absent in controls, but was detected in six patients with a 6.2% observed frequency. The difference between patients and controls was not statistically significant (p-value = 0.18). In male OA patients, the observed frequency of the TT genotype was higher (28.6%) in comparison to the 0% of the male controls (p-value = 0.1). The frequency of the G allele was 0.82 in controls and 0.78 in OA patients (p-value = 0.53).

Conclusions

The present study demonstrates that the 954G/C, -1026C/A, -1056G/T and -1173C/T SNPs of the NOS2A gene are not a risk factor for primary knee OA in Greek population. Moreover, -954G/C, -1026C/A and -1173C/T are rare, if not completely absent, in the Greek population. Additional research is mandatory in order to investigate the association of these SNPs with OA in different ethnic populations.

## Introduction

Osteoarthritis (OA) is a chronic degenerative joint condition, characterized by degradation of articular cartilage, synovial inflammation, osteophyte formation and subchondral bone sclerosis [1]. OA is a complex disease, influenced by a plethora of genetic and environmental factors, involving several molecular pathways [2,3]. Recently, nitric oxide (NO) has been reported to have a distinctive role in OA pathogenesis [4].

NO is a free radical produced by a reaction between L-arginine and oxygen, catalyzed by a family of enzymes named nitric oxide synthases (NOS) [5]. The inducible form of NOS (iNOS) is mainly generated by human chondrocytes and produces a sustained and extensive quantity of NO which exerts multiple roles in the articular cartilage: mediation of inflammation, chondrocyte apoptosis, extracellular matrix (ECM) degradation, synovial inflammation and subchondral bone dysfunction [6,7].

The elevated levels of iNOS and NO in arthritic tissues suggest that these tissues are actively generating NO [8,9]. On the contrary, the chondrocytes of patients without OA do not express iNOS, while iNOS knockout mice do not develop experimental OA, raising the possibility that the regulation of NO production may represent a new approach to the management of primary OA [7,10].

The gene encoding iNOS (NOS2A) is located on chromosome region 17q11.2-1q12, spans 37 Kb and consists of 26 exons [11]. Three single nucleotide polymorphisms (SNPs) in the promoter region, the -1173C/T (rs9282799), the -1026 C/A (rs2779249) and the -954G/C (rs1800482) are linked to increased iNOS expression, resulting in higher NO production [12]. The purpose of this study is to investigate the potential association between the aforementioned SNPs in the promoter of the NOS2A gene and the incidence of knee OA in Greek population.

## Materials and methods

After approval of the hospital’s ethics review committee, recruitment of participants for this case-control study took place between March 2015 and October 2016. The patient group included adults, suffering from knee OA, with a radiological score ≥ 2 in the Kellgren and Lawrence scale [13]. Patients were excluded when other causes of knee diseases existed, such as rheumatoid arthritis, post-traumatic arthritis, post-septic arthritis, osteonecrosis, skeletal dysplasia or congenital knee disorders. Control group included patients with no symptoms or clinical signs of OA, with a score < 2 in the Kellgren and Lawrence scale. Signed informed consent was obtained from all participants.

A total of 5 ml of peripheral blood were collected from each participant and stored into sodium citrate tubes at -20°C. DNA was extracted with a DNA extraction kit (QIAamp DNA Blood Midi Kit, QIAGEN Inc., Valencia, CA) according to manufacturer’s instructions. The isolated DNA was amplified by polymerase chain reaction (PCR) using the sense primer 5’-TTAACTTGGGAAAGACAAGAAGG-3’ and the antisense primer 5’- TCTGAACTAGTCACTTGAGG-3’ following an initial denaturation step at 95°C for 5 min and an amplification of 34 cycles in a three-step reaction that included denaturation at 94°C for 30 sec, annealing at 58°C for 30 sec and extension at 72°C for 2 min, with a final extension step at 72°C for 10 min. PCR was performed in a total volume of 24 μl (1 μl of DNA, 12.5 μl HotStart Taq Plus master mix, 9 μl H2O, and 1 μl of each primer) placed in 96-well PCR plates. The amplified fragments were analysed on the ABI 3500 Genetic Analyser (Applied Biosystems, Foster City, CA) with the use of the BigDye Terminator v3.1 kit (Applied Biosystems). The identification of polymorphic regions was determined with BioEdit software (Tom Hall, Ibis Therapeutics, Carlsbad, CA).

For the comparison of continuous values among cases and controls and among any other subgroups, the student’s unpaired t test was used. Categorical variables, such as gender and genotype frequencies, were compared using the chi-square (X^2^) test. A probability p-value less than 0.05 was considered statistically significant. Statistical analysis was performed using the PASW 18 (SPSS release 18.0; SPSS Inc., Chicago, IL).

## Results

**Figure 1:**
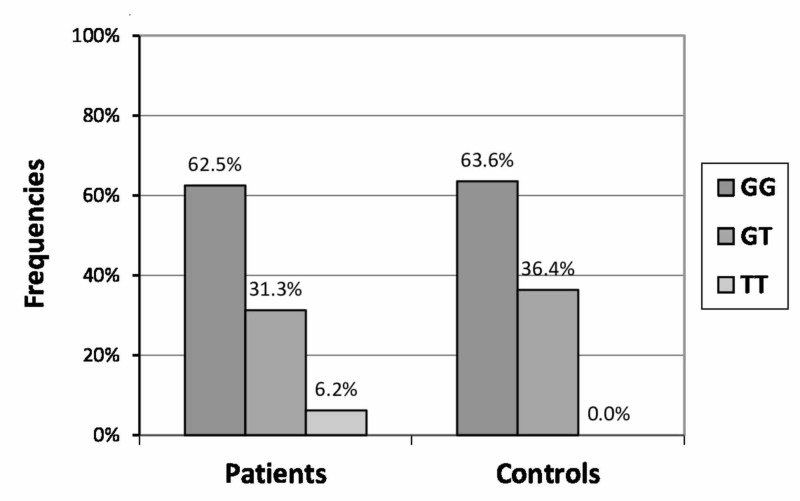
Genotype frequencies of -1056GT polymorphism in patients and controls

The study included 96 patients with primary knee OA and 44 control subjects. Demographics of the studied population are summarized in Table 1. The genotypic and allelic frequencies of the -1173C/T, -1026 C/A and -954G/C polymorphisms are summarized in Table 2. Only the common aforementioned SNPs were identified in the studied population, either in patients or controls, raising the possibility that the minor allele of these SNPs is completely absent in the Greek population. However, another SNP was identified at the site -1056G>T at the promoter region, where the initial G allele was substituted by the T allele. In about the 1/3 of the studied population, the T allele was present. Interestingly, the TT genotype was completely absent in controls, but was detected in six patients with a 6.2% observed frequency. The difference between patients and controls was not statistically significant (p-value = 0.18). In male OA patients, the observed frequency of the TT genotype was higher (28.6%) in comparison to the 0% of the male controls (p-value = 0.1). The frequency of the G allele was 0.82 in controls and 0.78 in OA patients (p-value = 0.53). Figure 1 shows the genotype frequencies of -1056G/T polymorphism in patients and controls.

**Table 1:** Descriptive characteristics of the study sample BMI: Bone Mass Index; SD: Standard Deviation.

	Controls	Cases	Overall	
	N (%)	N (%)	N (%)	p-value
Gender				0.062
Female	32 (72.7)	82 (85.4)	114 (81.4)	
Male	12 (27.3)	14 (14.6)	26 (18.6)	
Age (years)				0.0001
<71	34 (45.9)	40 (54.1)	74 (52.8)	
71<	10 (15.2)	56 (84.8)	66 (47.2)	
BMI				0.0008
<29	30 (50.0)	30 (50.0)	60 (47.6)	
29<	14 (21.2)	52 (78.8)	66 (52.4)	
	Mean (SD)	Mean (SD)	Mean (SD)	p-value
Age (years)	67.3 (10.3)	72.9 (9.3)	71.2 (9.9)	<0.001
BMI	27.5 (4.1)	30.6 (4.3)	29.5 (4.4)	<0.001

**Table 2:** Genotype and allelic frequencies

	Controls	Cases	Overall	
	N (%)	N (%)	N (%)	p-value
-954GC polymorphism				
GG	44 (100.0)	96 (100.0)	140 (100.0)	1.000
GC	0 (0.0)	0 (0.0)	0 (0.0)	
CC	0 (0.0)	0 (0.0)	0 (0.0)	
G allele frequency	1.00	1.00	1.00	1.000
C allele frequency	0.00	0.00	0.00	
-1026CA polymorphism				
CC	44 (100.0)	96 (100.0)	140 (100.0)	1.000
CA	0 (0.0)	0 (0.0)	0 (0.0)	
AA	0 (0.0)	0 (0.0)	0 (0.0)	
C allele frequency	1.00	1.00	1.00	1.000
A allele frequency	0.00	0.00	0.00	
-1056GT polymorphism				
GG	28 (63.6)	60 (62.5)	88 (62.8)	1.000
GT	16 (36.4)	30 (31.3)	46 (32.9)	0.565
TT	0 (0.0)	6 (6.2)	6 (4.3)	0.177
G allele frequency	0.82	0.78	0.79	0.528
T allele frequency	0.18	0.22	0.21	
-1173CT polymorphism				
CC	44 (100.0)	96 (100.0)	140 (100.0)	1.000
CT	0 (0.0)	0 (0.0)	0 (0.0)	
TT	0 (0.0)	0 (0.0)	0 (0.0)	
C allele frequency	1.00	1.00	1.00	1.000
T allele frequency	0.00	0.00	0.00	

## Discussion

To the best of our knowledge, this is the first study attempting to assess the role of the polymorphisms of the promoter of the NOS2A gene in the pathogenesis of knee OA. The results of this study suggest that for the -1173C/T, -1026 C/A and -954G/C polymorphisms, only the major allele is present, in the Greek population. The -1056G/T polymorphism was more common in OA patients, but without statistical significance.

Increased NO release and iNOS expression from osteoarthritic tissues has been widely reported [14]. NO mediates joint inflammation in OA, enhances ECM degradation, decreases ECM production and induces chondrocyte apoptosis [5, 15-17]. In the synovium, NO reduces the survival of OA synoviocytes and induces OA synovial fibroblast apoptosis [18-20].

The presence of SNPs in NOS2A gene has a distinctive role in several conditions affecting different populations. SNPs in the promoter region may influence the level of gene product. iNOS -1173 C/T polymorphism has been correlated with the sickle-cell disease in African populations. The C to T change creates a new sequence recognition site for the GATA-1 or GATA-2 transcription factors. The binding of these factors to specific DNA sequences may account for an increased degree of transcription from the -1173 C>T promoters [21]. iNOS variant rs2779249 (-1026 C>A) is also located in the promoter region of the gene and it has been shown that nucleotide change from C to A increases iNOS promoter transcriptional activity to five-fold leading to higher NO production [22]. This SNP has been associated with hypertension in Chinese Han population and with rheumatoid arthritis in Indians [23,24]. For the -954G>C polymorphism, the substitution of G to C results in a phenotype with a seven-fold higher baseline NOS activity [25]. This SNP has been studied in several pathologic conditions, including malaria, diabetes, rheumatoid arthritis, osteomyelitis and asthma [25-29].

In our study, genotyping of -954G>C, -1026C>A and -1173C>T in patients with knee OA and healthy controls revealed the presence of only the major allele of the aforementioned SNPs. Our findings are in agreement with the studies of other Caucasian populations. These SNPs seem to be ethnic-specific for the African population according to previous studies [30]. The -954 G>C variation has always been shown to be non-polymorphic in Caucasian population, all individuals carrying the wild-type G allele.

Our study revealed that the -1056G>T polymorphism of the NOS2A gene does not affect knee OA susceptibility in the Greek population. The observed lack of association may simply reflect that this SNP has a minor or no role in the onset of knee OA. Another possibility is that the specific polymorphism may contribute to the onset of knee OA, but this influence is too small to be detected with our study size, and a larger sample may be required. The sample size of the present study was not big enough, limiting statistical power for the detection of any existing association.

## Conclusions

Our study indicates that 954G>C, -1026C>A, -1056G>T and -1173C>T of the NOS2A gene are not a risk factor for the development of primary knee OA in Greek population. Moreover, for the -954G>C, -1026C>A and -1173C>T SNPs, the minor allele is rare, if not completely absent, in the Greek population. Additional research is necessary in order to give a global view of these SNPs in the pathogenesis of OA.
